# Impact of acupuncture on mortality in patients with disabilities and newly diagnosed heart failure: a nationwide cohort study

**DOI:** 10.3389/fmed.2025.1519588

**Published:** 2025-01-29

**Authors:** Hyungsun Jun, Dasol Park, Jae-Uk Sul, Moon Joo Cheong, Haerim Kim, Inae Youn, Jungtae Leem

**Affiliations:** ^1^Department of Diagnostics, College of Korean Medicine, Wonkwang University, Iksan, Republic of Korea; ^2^Dongshin University Gwangju Korean Medicine Hospital, Gwangju, Republic of Korea; ^3^Department of Medical Counseling, College of Health Sciences, Wonkwang University, Iksan-si, Republic of Korea; ^4^Department of Statistics, University of Seoul, Seoul, Republic of Korea; ^5^Department of Acupuncture and Moxibustion, National Medical Center, Seoul, Republic of Korea; ^6^Department of Integrated Medicine, Wonkwang University Korean Medicine Hospital, Iksan-si, Republic of Korea; ^7^Research Center of Traditional Korean Medicine, College of Korean Medicine, Wonkwang University, Iksan, Republic of Korea

**Keywords:** people with disabilities, heart failure, acupuncture, mortality, retrospective cohort study

## Abstract

**Objective:**

People with disabilities have high rates of cardiovascular diseases and mortality, and heart failure can worsen their condition. Therefore, preventing and managing cardiovascular diseases is particularly important for this population. Although acupuncture has been used for heart failure, research on its impact on mortality is limited. Given the unique pathophysiological characteristics of people with disabilities, this study aimed to evaluate the effect of acupuncture on mortality in those newly diagnosed with heart failure.

**Methods:**

This retrospective cohort study used data from the Korean National Health Insurance Service, focusing on people with disabilities diagnosed with heart failure between 2014 and 2016. Acupuncture exposure within 1 year of diagnosis was assessed by dividing the participants into acupuncture-exposed and non-exposed groups. Propensity score matching (PSM) was used to adjust for group differences, and all-cause mortality was tracked for 3 years. Cox proportional hazard models were employed to calculate hazard ratios (HRs) and confidence intervals (CIs). Dose-response relationships were also analyzed by dividing acupuncture frequency into quartiles.

**Results:**

After PSM, 21,001 individuals were included in both groups. The acupuncture-exposed group had a 20% lower risk of all-cause mortality (adjusted HR 0.80, 95% CI 0.76–0.84) than those in the non-exposed group. Higher acupuncture doses were associated with a greater reduction in mortality, with the highest dose group showing a 36% lower risk (adjusted HR 0.64, 95% CI 0.58–0.69) than those in the non-exposed group. The subgroup analysis showed a consistent reduction in mortality across most groups, particularly in women, older adults, higher-income individuals, and those with severe disabilities.

**Conclusion:**

This study suggests that acupuncture exposure is associated with reduced mortality in people with disabilities who are newly diagnosed with heart failure. While several limitations exist, we highlight the potential role of acupuncture in managing cardiovascular diseases in this population and encourage further research to support healthcare policies.

## 1 Introduction

Globally, ~15 out of every 100 people have a disability, with two to four having severe disabilities. As people live longer, the likelihood of developing a disability increases with age, leading to a growing number of individuals with disabilities (1). People with disabilities have a higher incidence and mortality rates of cardiovascular diseases than those without disabilities (2), and the occurrence of heart failure is reported to increase disability rates (3, 4). This suggests a bidirectional relationship between cardiovascular diseases and disability. Particularly, individuals with disabilities tend to have more comorbidities than those without disabilities (5, 6), which increases the risk of polypharmacy (4). Polypharmacy, in turn, is associated with higher mortality rates (7) and an increased likelihood of readmission (8), indicating that heart failure, which often requires polypharmacy, can worsen clinical outcomes in people with disabilities. Prevention plays a crucial role in the healthcare of people with disabilities and is generally divided into three stages. Primary prevention aims to eliminate causes before disease onset, secondary prevention focuses on early detection to prevent progression and long-term impacts, and tertiary prevention seeks to preserve function and reduce complications after disease development (5). Therefore, when a disease occurs in people with disabilities, there is a need to reduce the risks of polypharmacy and complications through an integrative medical approach. This includes physical therapies, such as acupuncture, as part of tertiary prevention, in addition to standard treatments.

Heart failure is closely related to various factors such as age and comorbidities (9). Generally, it refers to a condition in which structural or functional abnormalities of the heart, often caused by coronary artery disease, hypertension, or valvular heart disease, lead to impaired ventricular filling or ejection. The prevalence of heart failure varies widely, ranging from 0.2% to 17.7%, depending on the country and study; however, the average prevalence reported in studies involving all adults is ~3.4% (10). According to the 2021 American Heart Association Statistical Update, the prevalence of heart failure in the United States is ~6 million, or 1.8% of the total population (11). In Korea, the age-standardized incidence of heart failure decreased from 2002 to 2020, but its prevalence increased from 0.77% to 2.58%, and the mortality rate from heart failure rose from 3.0 to 15.6 per 100,000 population (12). Mortality rates due to heart failure remain high, with a 5-year mortality rate of ~50% (11–14). Despite advancements in standard treatments, survival rates following a diagnosis of heart failure remain as low as those for cancer (15).

In East Asian Traditional Medicine, various treatments such as acupuncture, herbal medicine, and pharmacopuncture are commonly used for patients with heart failure (16), with acupuncture being a long-standing intervention. South Korea's healthcare system is dualized, allowing traditional Korean medicine to be covered by national health insurance, which improves patients' accessibility to treatment. As a result, approximately seven out of 10 adults have used traditional Korean medicine, and among them, 94.3% have received acupuncture treatments (17). Acupuncture is known to work in heart failure by regulating sympathetic nervous activity and maintaining parasympathetic activity, leading to vasodilation and improved peripheral blood flow (18). Additionally, excessive sympathetic activation contributes to heart failure through mechanisms such as oxidative stress, inflammatory responses, and cardiac remodeling (19). Acupuncture protects cardiomyocytes and reduces myocardial ischemia by suppressing sympathetic activity (20). Standard pharmacological treatments for heart failure include renin-angiotensin-aldosterone system (RAAS) inhibitors, beta-blockers, diuretics, sodium-glucose co-transporter 2 inhibitors, and digoxin. These drugs affect heart failure through various mechanisms, with RAAS inhibitors, beta-blockers, and digoxin specifically reducing sympathetic activity in cardiomyocytes (19). Acupuncture shares some of these mechanisms with standard medications, suggesting its potential effectiveness in treating heart failure. It has been reported that acupuncture alone or in combination with conventional medicine reduced intensive care unit stays and readmission rates in patients with acute heart failure (21). In patients with chronic heart failure, it has shown improvements in exercise capacity, quality of life, and hemodynamic parameters (21). Additionally, among individuals diagnosed with hypertension, those who were exposed to acupuncture had significantly lower rates of all-cause mortality, myocardial infarction, and cardiovascular-related deaths (22). These studies suggest that acupuncture has potential benefits as a non-pharmacological adjunct to standard treatment for heart failure and other cardiovascular disease that may lead to heart failure.

People with disabilities often have multiple comorbidities in addition to their primary disability-related conditions (5, 6). The more severe the disability, the more negatively it impacts life expectancy (23), resulting in a shorter life expectancy compared with individuals without disabilities (24). Therefore, when a high-mortality disease such as heart failure occurs in people with disabilities, the standard treatment guidelines designed for individuals without disabilities may be insufficient, and an integrative medical approach that considers disabilities is necessary. However, conducting clinical trials on people with disabilities is challenging, leading to a knowledge gap regarding effective treatments. Similarly, research on the use of acupuncture in people with disabilities is lacking, making it difficult to strongly recommend its use owing to insufficient evidence. This study aims to address this gap by conducting a retrospective cohort study using data from the Korean National Health Insurance Service (NHIS). The goal is to analyze the real-world impact of acupuncture exposure on mortality in people with disabilities and newly diagnosed heart failure across the Korean population.

## 2 Methods

### 2.1 Data sources

The Korean NHIS has established a national health information database to support policy and academic research. As of March 2024, the NHIS covers 51,417,000 subscribers, representing over 99% of the total population, making it one of the largest healthcare systems in the world. The data can be accessed through the National Health Insurance Data Sharing Service website (http://nhiss.nhis.or.kr). The research team obtained customized data containing health insurance claims for registered individuals with disabilities in Korea from 2013 to 2019 (NHIS-2021-1-301). The data included demographic characteristics, medical histories, health behaviors, physical measurements, test results, medical utilization, mortality, and long-term care services for older individuals. This study was approved by the Institutional Review Board of the Dongshin University Korean Medicine Hospital (DSGOH_E_2021_003) and was conducted in accordance with the principles of the Declaration of Helsinki.

### 2.2 Study participants

**Figure 1:**
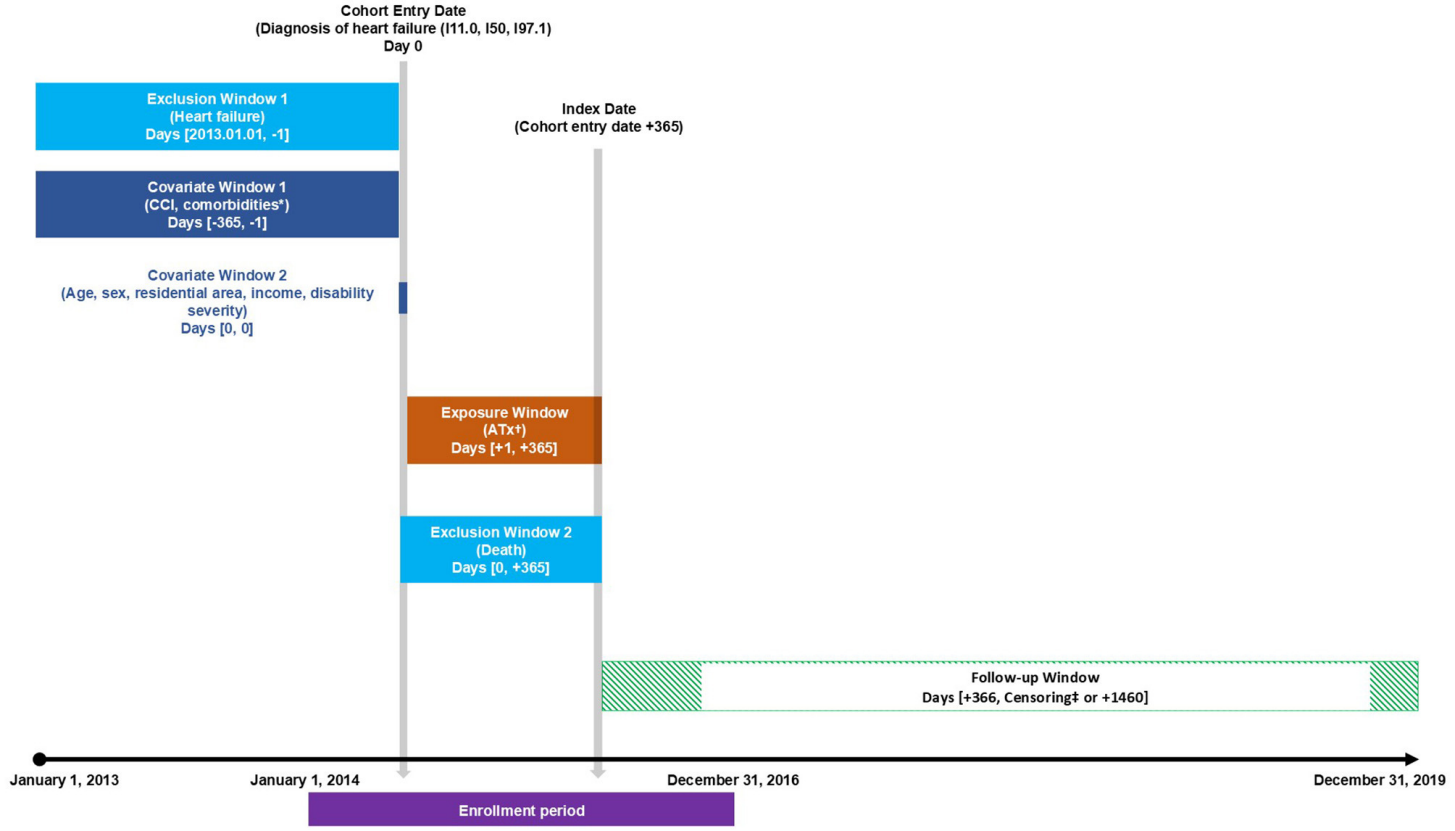
Study design. ATx, acupuncture treatment; CCI, Charlson Comorbidity Index. *Prespecified comorbidities include hypertension, diabetes mellitus, hyperlipidemia, ischemic stroke, chronic obstructive pulmonary disease, atrial fibrillation, peripheral artery disease, chronic kidney disease, and chronic liver disease. ^†^Assessment of whether the patient received acupuncture treatment two or more times or not at all. ^‡^Censored at the earliest occurrence of death or 3 years from the start of follow-up.

The study design and plan are presented in Figure 1. The individuals classified as disabled in the NHIS dataset were those registered as having disabilities according to the Korean Act on Welfare of Persons with Disabilities, which defines disability as “a physical or mental impairment that significantly restricts daily or social activities over an extended period.” The NHIS dataset includes information on disability registration, types, and severity, and as of 2014, disabilities were categorized into non-disabled, physical disability, brain lesion disability, visual impairment, hearing impairment, speech impairment, intellectual disability, mental disability, and other disabilities (including renal, autism, cardiac, respiratory, hepatic, facial, stoma, and epilepsy-related disabilities) (25). Only patients with disabilities who were newly diagnosed with heart failure between 1 January 2014, and 31 December 2016 were included. Heart failure was defined based on the International Classification of Diseases, 10th Revision (ICD-10) codes I11.0 (hypertensive heart disease with [congestive] heart failure), I50 (heart failure), and I97.1 (other functional disturbances following cardiac surgery) as per the guidelines of the Korean Health Insurance Review and Assessment Service (26). To qualify as a patient with heart failure, the relevant ICD-10 codes had to be present in either the primary or secondary diagnosis. Additionally, the patient must have had at least two outpatient visits or one inpatient admission within 1 year of the initial diagnosis. A washout period was set for 2013 to ensure that only newly diagnosed patients were included, and the study was restricted to individuals aged 19 and older. The index date was set 1 year after the cohort entry date, which is the date of the first heart failure diagnosis, and only individuals who did not die within the 1st year after diagnosis were included in the follow-up.

The acupuncture exposure group was identified using the codes 40011 (acupuncture, single body part) and 40012 (acupuncture, multiple body parts). Patients who received acupuncture treatment at least twice within 1 year after their heart failure diagnosis were classified as the acupuncture treatment group, whereas those who did not receive acupuncture at all were defined as the conventional treatment group. Patients were assigned to their respective exposure groups regardless of the specific acupuncture points or diagnosis codes associated with their treatment.

### 2.3 Outcomes and covariates

The primary outcome of the study was all-cause mortality, and all patients were followed until death or up to 3 years from the index date. Mortality data were confirmed by linking unique identification numbers with the national death registration data provided by Statistics Korea.

The sociodemographic profiles in the Korean NHIS sample cohort data and the validity of operational definitions for cardiovascular-related comorbidities have been evaluated and utilized in numerous studies (27–29). These covariates and classification criteria were also employed in this study. The sociodemographic and health-related covariates were defined as follows. Age was categorized into the following groups: 20–29, 30–39, 40–49, 50–59, 60–69, 70–79, and 80 years or older. Sex was classified as male or female, and residence was divided into metropolitan, urban, or rural areas (30). Income was classified into medical aid, low, middle, and high categories (31). As of 2014, the severity of disabilities registered in Korea was assessed by nationally designated disability rating agencies. Disabilities were categorized into six grades (1–6) based on evaluations of medical conditions, functional status, and limitations in daily activities, with grades 1 and 2 classified as severe and grades 3–6 as mild according to administrative criteria. This classification was applied to evaluate disability severity on the cohort entry date (2, 25). The Charlson Comorbidity Index (CCI) was used to assess overall health status (32). The presence of comorbidities such as hypertension, diabetes, hyperlipidemia, ischemic stroke, chronic obstructive pulmonary disease (COPD), atrial fibrillation, peripheral artery disease, chronic kidney disease, and chronic liver disease was also evaluated based on data from 1 year before the cohort entry date (28). Detailed variable classifications and diagnostic definitions are provided in Supplementary Table S1.

### 2.4 Statistical analysis

To minimize baseline characteristic imbalances and the influence of confounding factors between the acupuncture and conventional treatment groups, propensity score matching (PSM) was employed. A multiple logistic regression model was used to calculate the propensity scores, representing the probability of each participant being assigned to a particular group based on the covariates. After calculating the propensity scores, matching was performed using a caliper width of 0.01 and the nearest-neighbor algorithm. A standardized mean difference of less than 0.1 was set as the acceptable threshold to evaluate the balance between the groups. Furthermore, Schoenfeld residuals were used to check for proportional hazard assumptions and to prevent bias due to imbalances between the acupuncture and conventional treatment groups.

Descriptive statistics are used to summarize the baseline characteristics. Categorical variables are presented as numbers and percentages (%). Overall mortality rates were calculated by dividing the number of events by person-years at risk and are reported as events per 1,000 person-years. Hazard ratios (HRs) and 95% confidence intervals were estimated using the Cox proportional hazards model. Kaplan–Meier curves were generated to visualize the survival probabilities between the acupuncture and conventional treatment groups. The results are presented before and after adjusting for age, sex, residence, income, disability severity, CCI, hypertension, and hyperlipidemia. To assess the dose-response relationship, the acupuncture group was divided into four quartiles, with the first quartile representing the group with the fewest acupuncture sessions and the fourth quartile representing the group with the most sessions. Visual and statistical tests were conducted to evaluate the dose-response effect. Additionally, subgroup analyses of the dose-response relationship were performed based on the covariates used in the survival analysis. Participants were stratified into subgroups by age (<60, 60–74, ≥75), sex (male, female), residence (urban, rural), income (low, middle, high), disability severity (mild, severe), CCI (<3, ≥3), and the presence of comorbidities. The results are presented using forest plots. A *p*-value of <0.05 is considered statistically significant in two-tailed tests. Statistical analyses were conducted using SAS version 9.4 (SAS Institute Inc., Cary, NC, USA) and R version 3.3.2 (The R Foundation, http://www.R-project.org). R packages including “survival,” “survminer,” “moonBook,” “ggplot2,” “survey,” “optmatch,” and “MatchIt” were used for analysis, visualization, and PSM.

## 3 Results

### 3.1 Patient characteristics

**Figure 2:**
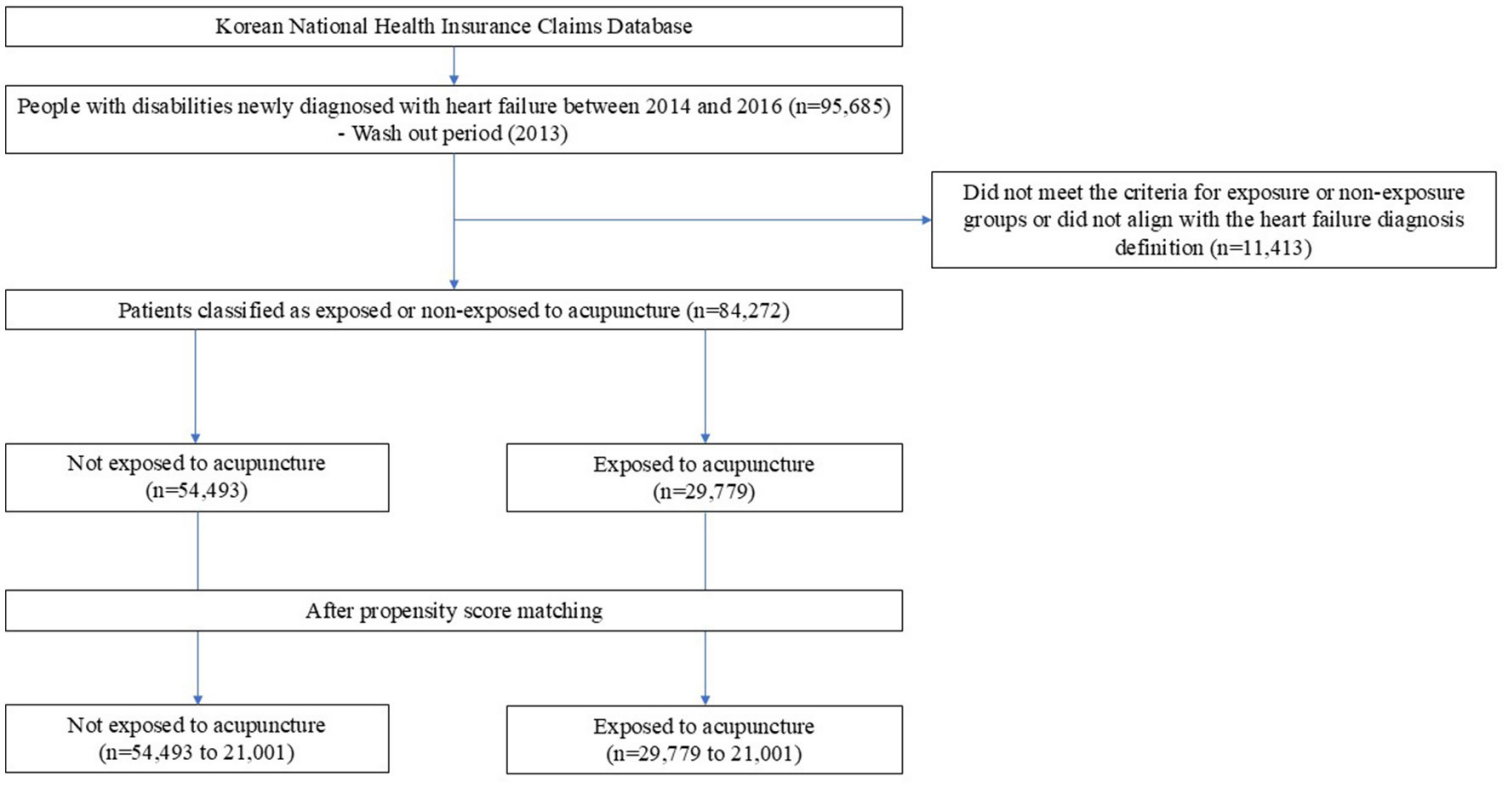
Study flowchart.

Between 2014 and 2016, a total of 95,685 individuals with disabilities were newly diagnosed with heart failure. After applying the inclusion and exclusion criteria, 84,272 patients with heart failure were included. The patients were divided into an acupuncture-exposed group with 29,779 individuals and a non-exposed group with 54,493 individuals based on their acupuncture exposure. After PSM, 21,001 patients were selected for each group (Figure 2). Before PSM, the acupuncture group had a higher proportion of older adults, women, rural residents, individuals with higher incomes, those with mild disabilities, higher CCI scores, and comorbidities, such as hypertension, diabetes, hyperlipidemia, stroke, and COPD, than those in the conventional treatment group. After PSM, the baseline characteristics between the two groups became comparable (Table 1).

**Table 1:** Baseline characteristics.

	**Before propensity score matching**				**After propensity score matching**			

	**Acupuncture (*****n*** = **29,779)**	**Conventional (*****n*** = **54,493)**	***p***	**SMD**	**Acupuncture (*****n*** = **21,001)**	**Conventional (*****n*** = **21,001)**	***p***	**SMD**
Age, years			< 0.001	0.297			1	< 0.001
20–29	42 (0.1)	335 (0.6)			13 (0.1)	13 (0.1)		
30–39	164 (0.6)	891 (1.6)			77 (0.4)	77 (0.4)		
40–49	666 (2.2)	2,768 (5.1)			383 (1.8)	383 (1.8)		
50–59	2,733 (9.2)	7,613 (14.0)			1,796 (8.6)	1,796 (8.6)		
60–69	6,482 (21.8)	12,269 (22.5)			4,446 (21.2)	4,446 (21.2)		
70–79	13,025 (43.7)	18,229 (33.5)			9,214 (43.9)	9,214 (43.9)		
≥80	6,667 (22.4)	12,388 (22.7)			5,072 (24.2)	5,072 (24.2)		
Sex			< 0.001	0.315			0.968	< 0.001
Male	11,192 (37.6)	28,930 (53.1)			7,898 (37.6)	7,903 (37.6)		
Female	18,587 (62.4)	25,563 (46.9)			13,103 (62.4)	13,098 (62.4)		
Residential area			< 0.001	0.078			1	< 0.001
Metropolitan	9,948 (33.5)	20,224 (37.2)			7,137 (34.0)	7,137 (34.0)		
Urban	5,738 (19.3)	9,822 (18.1)			3,467 (16.5)	3,467 (16.5)		
Rural	13,985 (47.1)	24,251 (44.7)			10,397 (49.5)	10,397 (49.5)		
Income			< 0.001	0.1			1	< 0.001
Medical aid	4,812 (16.2)	10,469 (19.2)			3,015 (14.4)	3,015 (14.4)		
Low	5,734 (19.3)	10,686 (19.6)			3,877 (18.5)	3,877 (18.5)		
Middle	7,131 (23.9)	13,448 (24.7)			5,058 (24.1)	5,058 (24.1)		
High	12,102 (40.6)	19,890 (36.5)			9,051 (43.1)	9,051 (43.1)		
Severity of disability			< 0.001	0.295			0.831	0.002
Severe	4,179 (14.0)	14,000 (25.7)			2,425 (11.5)	2,440 (11.6)		
Mild	25,600 (86.0)	40,493 (74.3)			18,576 (88.5)	18,561 (88.4)		
CCI score			< 0.001	0.128			1	< 0.001
0	4,234 (14.2)	9,950 (18.3)			3,858 (18.4)	3,858 (18.4)		
1	8,231 (27.7)	15,570 (28.7)			6,749 (32.1)	6,749 (32.1)		
2	7,000 (23.5)	12,013 (22.1)			5,128 (24.4)	5,128 (24.4)		
≥3	10,314 (34.6)	16,960 (30.9)			5,266 (25.1)	5,266 (25.1)		
Hypertension	21,164 (71.1)	36,622 (67.4)	< 0.001	0.08	15,473 (73.7)	15,473 (73.7)	1	< 0.001
DM	8,466 (28.5)	15,053 (27.7)	0.024	0.016	4,753 (22.6)	4,733 (22.5)	0.825	0.002
Dyslipidemia	7,545 (25.4)	12,065 (22.2)	< 0.001	0.074	4,135 (19.7)	4,109 (19.6)	0.759	0.003
Ischemic stroke	3,464 (11.6)	5,825 (10.7)	< 0.001	0.029	1,522 (7.2)	1,522 (7.2)	1	< 0.001
COPD	3681 (12.4)	5687 (10.5)	< 0.001	0.06	1776 (8.5)	1804 (8.6)	0.637	0.005
AF	1,916 (6.4)	3,606 (6.6)	0.267	0.008	663 (3.2)	663 (3.2)	1	< 0.001
PAD	605 (2.0)	1,023 (1.9)	0.139	0.011	151 (0.7)	136 (0.6)	0.407	0.009
CKD	1,698 (5.7)	6,716 (12.4)	< 0.001	0.234	680 (3.2)	660 (3.1)	0.598	0.005
CLD	1,238 (4.2)	2,171 (4.0)	0.26	0.008	279 (1.3)	279 (1.3)	1	< 0.001

AF, atrial fibrillation; CCI, Charlson Comorbidity Index; CKD, chronic kidney disease; CLD, chronic liver disease; COPD, chronic obstructive pulmonary disease; DM, diabetes mellitus; PAD, peripheral artery disease; SMD, standardized mean difference.

Baseline characteristics are presented as *n* (%).

### 3.2 All-cause mortality

**Figure 3:**
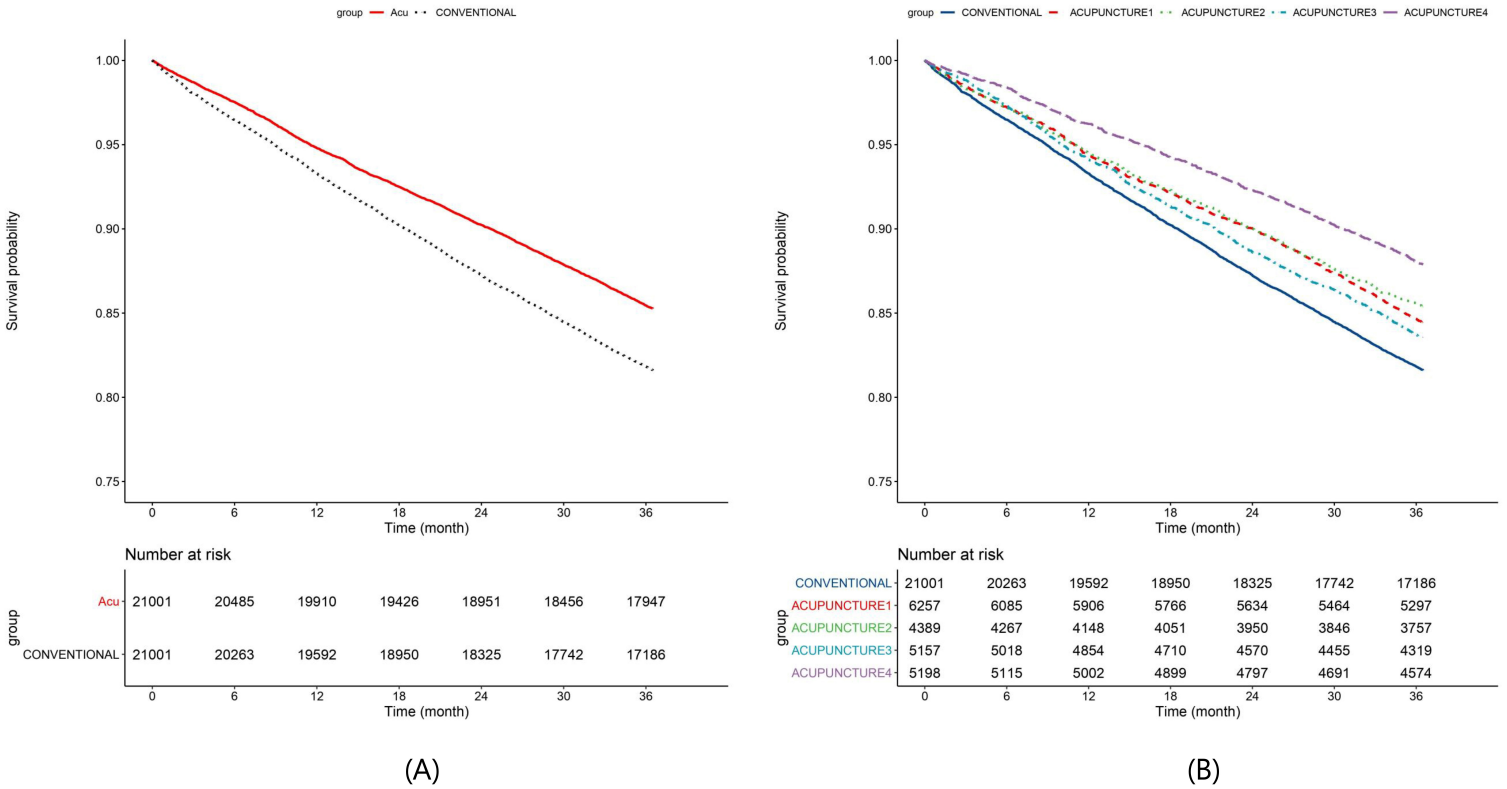
Kaplan–Meier curves. **(A)** Mortality, **(B)** mortality by acupuncture dose. The conventional group consists of individuals who have never received acupuncture treatment, whereas the acupuncture group comprises those who have undergone acupuncture at least twice within 1 year after their heart failure diagnosis. The acupuncture group is further categorized into four subgroups based on the number of treatments received: acupuncture 1 group (2–4 sessions), acupuncture 2 group (5–8 sessions), acupuncture 3 group (9–18 sessions), and acupuncture 4 group (more than 19 sessions).

All-cause mortality was lower in the acupuncture group compared with the conventional treatment group (53.07 per 1,000 person-years vs. 67.90 per 1,000 person-years; crude HR 0.78 [0.75–0.82]). After adjusting for age, sex, residence, income, disability severity, CCI, hypertension, and hyperlipidemia, the acupuncture group had a 20% lower risk of death compared with the conventional treatment group (adjusted HR 0.80 [0.76–0.84]) (Table 2). The Kaplan–Meier curve also showed that the acupuncture group had a higher survival probability from all-cause mortality than the conventional treatment group (Figure 3A).

**Table 2:** Incidence rates and hazard ratios for mortality.

	**Acupuncture sessions, *n***	**Total, *n***	**Events, *n* (%)**	**IR/1000 person-years**	**Crude HR (95% CI)**	Adjusted HR^*^(95% CI)
**All-cause mortality**						
Conventional	0	21,001	3,863 (18.39)	67.90	1 [Ref]	1 [Ref]
Acupuncture	≥2	21,001	3,093 (14.73)	53.07	0.78 (0.75–0.82)	0.80 (0.76–0.84)
Acupuncture 1	2–4	6,257	974 (15.57)	56.33	0.83 (0.77–0.89)	0.87 (0.81–0.93)
Acupuncture 2	5–8	4,389	642 (14.63)	52.84	0.78 (0.72–0.85)	0.81 (0.75–0.88)
Acupuncture 3	9–18	5,157	848 (16.44)	59.90	0.88 (0.82–0.95)	0.88 (0.81–0.94)
Acupuncture 4	≥19	5,198	629 (12.10)	42.84	0.63 (0.58–0.69)	0.64 (0.58–0.69)

CI, confidence interval; HR, hazard ratio; IR, incidence rate.

^*^Adjusted for age, sex, disability severity, income, residence area, hypertension, dyslipidemia, and Charlson Comorbidity Index.

### 3.3 Relationship between acupuncture dose and mortality

To assess the change in mortality based on the number of acupuncture sessions, the acupuncture group was divided into four subgroups: acupuncture 1 (2–4 sessions), acupuncture 2 (5–8 sessions), acupuncture 3 (9–18 sessions), and acupuncture 4 (≥19 sessions). Compared with the conventional treatment group, the acupuncture 1 group had a 13% lower risk of death (adjusted HR 0.87 [0.81–0.93]), whereas the acupuncture 4 group showed the largest reduction in mortality risk, with a 36% decrease (adjusted HR 0.64 [0.58–0.69]) (Table 2). The Kaplan–Meier curve also showed the highest survival probability in the acupuncture 4 group, which received the highest number of sessions (Figure 3B). Subgroup analyses consistently demonstrated lower mortality rates in most groups than in the conventional treatment group, with the lowest HRs observed in the acupuncture 4 group. However, significant mortality reduction was not observed in subgroups such as those under 60 years of age or those with atrial fibrillation, peripheral artery disease, chronic kidney disease, or chronic liver disease when compared with the conventional treatment group. Furthermore, the mortality risk was lower in older individuals, high-income groups compared with those in low-income groups, those with severe disabilities compared with those with mild disabilities, and in women compared with men. The absence of comorbidities, except hypertension, diabetes, and ischemic stroke, was also associated with a lower risk of death (Supplementary Figure S1).

## 4 Discussions

### 4.1 Summary of findings

In this study, we analyzed the difference in mortality within 3 years between people with disabilities newly diagnosed with heart failure from 2014 to 2016, comparing those who received at least two acupuncture treatments within 1 year of diagnosis with those who did not. The results showed that the acupuncture-exposed group had a significantly lower all-cause mortality rate than the non-exposed group. The Kaplan–Meier analysis also demonstrated higher survival rates in the acupuncture group than in the non-exposed group. Moreover, the dose-response analysis revealed that the group receiving the highest number of acupuncture sessions had the lowest mortality rate. Most subgroups also exhibited lower mortality risks in the acupuncture group. These findings suggest that acupuncture may contribute to reducing mortality in people with disabilities who are diagnosed with heart failure.

### 4.2 Review of the efficacy of acupuncture treatment in individuals with disabilities and heart failure

Acupuncture can benefit patients with acute and chronic heart failure, including improvements in hemodynamic parameters (21, 33). Moreover, a previous cohort study conducted by our research team using sample data from the Korean NHIS found that patients with heart failure, regardless of disability status, had a 27% lower risk of all-cause mortality when exposed to acupuncture than when not exposed (Unpublished data). A 2024 systematic review by Ganglani et al. (34) concluded that acupuncture may help manage dyspnea and heart failure. People with disabilities tend to have a higher prevalence of chronic diseases and risk factors such as hypertension, diabetes, hyperlipidemia, and obesity, which can negatively affect the incidence of cardiovascular diseases and related mortality (35). In this context, acupuncture treatment for individuals with cardiovascular diseases, including those with disabilities, is likely to yield positive effects similar to those observed in individuals without disabilities. The findings of this study further support the potential benefits of acupuncture in patients with heart failure and disabilities.

In South Korea, individuals are categorized into 15 disability types based on the Act on Welfare of Persons with Disabilities (36). These disabilities are broadly divided into physical and mental. Physical disabilities include external functional impairments, such as mobility impairments, brain lesions, visual impairments, hearing impairments, speech impairments, and facial disfigurement, as well as disabilities related to internal organs. Mental disabilities include developmental disabilities and mental disorders. According to the 2015 Korea Disability Statistics Yearbook, as of 2014, ~2.49 million people were registered as having disabilities, with 87.9% having physical disabilities. Among them, mobility impairments accounted for the largest proportion at 51.9%, followed by brain lesions, visual impairments, and hearing impairments, each at 10.1% (37). People with physical disabilities have been reported to have a higher prevalence of chronic conditions such as hypertension and diabetes and are at a greater risk of coronary artery disease than those with non-physical disabilities. This suggests that the pathophysiology may differ depending on the type of disability (38–40). Individuals with limited physical activity owing to disabilities have higher cardiovascular mortality rates, whereas those who maintain higher physical activity levels despite having disabilities tend to have lower cardiovascular-related mortality (41). When heart failure coexists with physical disabilities, further limitations in physical activity due to declining heart function may lead to worse outcomes. Therefore, enhancing the physical activity of individuals with physical disabilities may positively affect their prognosis. Acupuncture may improve the physical activity of patients with physical disabilities. Wang et al. (42) demonstrated that acupuncture improved motor impairments in a dose-dependent manner in patients with physical disabilities due to stroke, thereby enhancing their quality of life and independence. Furthermore, Yang et al. (43), through a review of 31 trials, confirmed that acupuncture improved independence, reduced overall neurological deficits, and mitigated specific neurological impairments during the rehabilitation of patients with a history of stroke.

Individuals with intellectual disabilities have been found to have a higher prevalence of obesity, hypertension, hyperglycemia, and hyperlipidemia than the general population without intellectual disabilities (44). In a Danish cohort study by Wang et al. (45), individuals with intellectual disabilities had higher rates of cerebrovascular disease, heart failure, hypertension, and deep vein thrombosis than those without intellectual disabilities. This indicates that the risk of cardiovascular disease is also elevated in those with mental disabilities like intellectual disabilities. Zwack et al. (46) reported that young adults with intellectual disabilities are more likely to have cardiovascular and metabolic risk factors and that autonomic nervous system dysfunction is associated with the severity of their disability. This suggests that autonomic nervous system function can play a crucial role in reducing cardiovascular risk in adults with intellectual disabilities. Acupuncture has been shown to help regulate sympathetic nervous system activity while maintaining parasympathetic activity (18). This has been reported to lower blood pressure and heart rate by suppressing sympathetic excitation in patients with severe heart failure under mental stress (47, 48). Therefore, acupuncture may have beneficial cardiovascular effects in patients with heart failure and intellectual disabilities by regulating the autonomic nervous function.

This study confirmed a significant association between acupuncture exposure and lower mortality rates in individuals with disabilities diagnosed with heart failure compared with those in the non-exposed group. The observed effects may be attributed to the mechanisms of acupuncture, such as the improvement of motor dysfunction and regulation of autonomic nervous function. However, given that different types of disabilities present heterogeneous risk factors and outcomes for cardiovascular diseases, the acupuncture effects may also vary depending on the type of disability; therefore, the results of this study should be interpreted with caution. Currently, research on the epidemiology and pathophysiology of cardiovascular diseases in people with disabilities and the effects and mechanisms of various interventions, including acupuncture, is limited. Further studies that account for the pathophysiological differences across disability types are needed to clarify the effectiveness and efficacy of acupuncture. Moreover, this study found that patients receiving more than 19 acupuncture sessions had the lowest mortality rates. Although disability severity was analyzed by dividing it into mild and severe categories, this classification does not necessarily reflect the degree of physical activity limitation caused by disability. Therefore, the high-dose acupuncture group might have been influenced by their level of physical activity or mobility. To address this issue, extensive basic and clinical research is required to examine the cardiovascular burden associated with different types of disabilities, along with studies that incorporate the level of physical activity into their design and execution. The findings of such future studies can contribute to the development of clinical guidelines for optimally treating and managing cardiovascular diseases in people with disabilities, including acupuncture as part of integrative care. These studies can also serve as important resources for formulating healthcare policies aimed at addressing the specific needs of people with disabilities.

In addition to the groups classified by the frequency of acupuncture treatments, most subgroups showed significantly lower mortality risks in the acupuncture group compared to the general treatment group. However, a significant reduction in mortality associated with acupuncture was not observed in males, individuals under 60 years of age, or those with mild disabilities. Similarly, consistent results were not found in groups with CCI score of 3 or higher, or in patients with stroke of COPD. These findings may be attributed to the relatively small sample sizes in these subgroups. For patients with peripheral artery disease, chronic kidney disease, or chronic liver disease, no significant reduction in mortality was observed. While limited sample sizes could partly explain these findings, the severity of these conditions and their high associated mortality risk likely influenced the outcomes. Future research should aim to secure sufficient sample sizes and include analyses that account for the severity of heart failure and comorbidities to provide a more detailed evaluation of the effects of acupuncture.

### 4.3 Proposal for future research

Future research should consider that the causes of mortality vary significantly depending on the type of disability (49). It is essential to conduct further studies to determine which types of disabilities benefit the most from acupuncture in managing cardiovascular diseases. Additionally, exploring the mechanisms by which acupuncture influences outcomes is necessary. In particular, individuals with mobility limitations may benefit from acupuncture but may face challenges in accessing such treatments. To address this, policy considerations such as home-based acupuncture care should be explored. These policies should be supported by high-quality evidence on the effectiveness of acupuncture, along with cost-effectiveness evaluations. Moreover, this study did not consider medication use at the time of heart failure diagnosis. Future studies should consider performing PSM that includes medication use to provide a more comprehensive analysis.

### 4.4 Strengths and limitations

Research on individuals with disabilities is considerably lacking, leading to a substantial knowledge gap compared with studies on non-disabled populations. This study is valuable in that it investigated the effects of acupuncture exposure in individuals diagnosed with heart failure, confirming the potential applicability of acupuncture in managing cardiovascular diseases in people with disabilities. We highlight the need for further research and identify key considerations for studies involving populations with disabilities. Additionally, this study is notable as it is the first to evaluate the impact of acupuncture on heart failure in individuals with disabilities using a large-scale dataset of 2.5 million people, employing methods such as PSM to improve comparability across heterogeneous disabled populations. However, this study has several limitations. First, the analysis included only those who survived for 1 year after their initial heart failure diagnosis, excluding patients who died within the 1st year. Therefore, individuals with more severe conditions might have been omitted from the analysis. This design was implemented not only to assess long-term outcomes but also to avoid potential distortions in the analysis caused by the acute treatment effects if patients who died shortly after the initial diagnosis were included. Further studies are needed to clarify the effects of acupuncture on individuals with more severe conditions. Second, while we stratified participants by the severity of their disabilities, the disability severity might not have fully reflected physical activity levels, a key risk factor for cardiovascular disease, potentially introducing bias. This limitation stems from the administrative nature of NHIS data, which does not provide quantitative data on physical activity levels. Future studies should address this limitation using appropriate study designs. Third, the frequency of acupuncture treatments might have varied based on disability type or mobility; however, this study did not conduct a detailed analysis considering specific disability types. NHIS data primarily categorize disability types for administrative purposes, making it difficult to objectively reflect characteristics related to activity levels and cardiovascular diseases in specific disability types, such as sensory, physical, and neuropsychiatric disabilities. Additionally, this study could not assess the effects of acupuncture exposure on indicators directly related to cardiac function and prognosis, such as objective hemodynamic measures, functional capacity assessments, or hematological markers. Future studies should address this issue by using data sources that include relevant clinical indicators. Fourth, although the course and pathophysiology of heart failure may vary depending on the type of disability present before the heart failure diagnosis, this study did not make such distinctions. Fifth, while this study categorized acupuncture exposure into four groups based on quartiles to analyze differences in outcomes and explore dose-response relationships, this grouping was arbitrary and did not account for the duration of treatment. As a result, clinically meaningful treatment dosage groups were not established. These findings should therefore be interpreted with caution. There is currently no prior research on clinically meaningful acupuncture dosages for individuals with disabilities and heart failure. Further studies are needed to build upon these results and determine effective treatment dosages. Sixth, this study was designed to evaluate the impact of acupuncture exposure in heart failure patients, without distinguishing whether the acupuncture treatments were specifically intended for heart failure management. This design does not allow for a direct assessment of the efficacy of acupuncture in treating heart failure, and the findings should therefore be interpreted with caution. To address this limitation, clinical studies targeting disabled population diagnosed with heart failure should be conducted to evaluate the effects of acupuncture interventions specifically designed for heart failure treatment.

## 5 Conclusion

This study confirmed that acupuncture exposure was associated with reduced all-cause mortality in individuals diagnosed with heart failure, demonstrating a dose-response relationship in which higher frequencies of acupuncture treatment were linked to lower mortality risks. These findings suggest that acupuncture could potentially serve as a valuable therapeutic tool for managing cardiovascular diseases in individuals with disabilities and may have a broader positive impact on this population. However, this study was some limitations, including the exclusion of patients who died within the 1st year, which may have resulted in the omission of those with severe conditions, and the inability of disability severity to fully reflect physical activity levels. To address these limitations, future studies should utilize clinical data that objectively and quantitatively measure variables such as disability type, physical activity levels, and the severity of heart failure. Such research would benefit from robust study designs and analyses that incorporate these factors.

## Data Availability

Publicly available datasets were analyzed in this study. This data can be found at: The NHIS provided the data for this investigation (NHIS-2021-1-301). Data sharing is limited to comply with privacy requirements, and the NHIS forbids the transfer, renting, or sale of datasets to other organizations. Researchers can request NHIS data from their official website (https://nhiss.nhis.or.kr) if granted access.

## References

[1] Okoro CA Hollis ND Cyrus AC Griffin-Blake S. Prevalence of disabilities and health care access by disability status and type among adults - United States, 2016. MMWR Morb Mortal Wkly Rep. (2018) 67:882–7. 10.15585/mmwr.mm6732a3 30114005 PMC6095650

[2] Son KY Kim SH Sunwoo S Lee J-Y Lim S Kim YS. Association between disability and cardiovascular event and mortality: a nationwide representative longitudinal study in Korea. PLoS ONE. (2020) 15:e0236665. 10.1371/journal.pone.0236665 32730313 PMC7392251

[3] Dunlay SM Manemann SM Chamberlain AM Cheville AL Jiang R Weston SA. Activities of daily living and outcomes in heart failure. Circ Heart Fail. (2015) 8:261–7. 10.1161/CIRCHEARTFAILURE.114.001542 25717059 PMC4366326

[4] Wong CY Chaudhry SI Desai MM Krumholz HM. Trends in comorbidity, disability, and polypharmacy in heart failure. Am J Med. (2011) 124:136–43. 10.1016/j.amjmed.2010.08.017 21295193 PMC3237399

[5] World Health Organization. World Report on Disability 2011. Geneva. (2011). Available at: https://www.who.int/teams/noncommunicable-diseases/sensory-functions-disability-and-rehabilitation/world-report-on-disability (accessed 12 August 2024).

[6] Rana MS Alam MB Khanam SJ Kabir MI Khandaker G Khan MN. Prevalence and patterns of comorbidities in people with disabilities and their associated socio-demographic factors. Sci Rep. (2024) 14:1425. 10.1038/s41598-024-51678-4 38228776 PMC10791601

[7] Leelakanok N Holcombe AL Lund BC Gu X Schweizer ML. Association between polypharmacy and death: a systematic review and meta-analysis. J Am Pharm Assoc JAPhA. (2017) 57:729–38.e10. 10.1016/j.japh.2017.06.002 28784299

[8] Zaninotto P Huang YT Di Gessa G Abell J Lassale C Steptoe A. Polypharmacy is a risk factor for hospital admission due to a fall: evidence from the English Longitudinal Study of Ageing. BMC Public Health. (2020) 20:1804. 10.1186/s12889-020-09920-x 33243195 PMC7690163

[9] Savarese G Becher PM Lund LH Seferovic P Rosano GMC Coats AJS. Global burden of heart failure: a comprehensive and updated review of epidemiology. Cardiovasc Res. (2023) 118:3272–87. 10.1093/cvr/cvac013 35150240

[10] Emmons-Bell S Johnson C Roth G. Prevalence, incidence and survival of heart failure: a systematic review. Heart Br Card Soc. (2022) 108:1351–60. 10.1136/heartjnl-2021-320131 35042750 PMC9380485

[11] Roger VL. Epidemiology of heart failure: a contemporary perspective. Circ Res. (2021) 128:1421–34. 10.1161/CIRCRESAHA.121.318172 33983838

[12] Lee CJ Lee H Yoon M Chun K-H Kong MG Jung M-H. Heart failure statistics 2024 update: a report from the Korean society of heart failure. Int J Heart Fail. (2024) 6:56–69. 10.36628/ijhf.2024.0010 38694933 PMC11058436

[13] Tsao CW Lyass A Enserro D Larson MG Ho JE Kizer JR. Temporal trends in the incidence of and mortality associated with heart failure with preserved and reduced ejection fraction. JACC Heart Fail. (2018) 6:678–85. 10.1016/j.jchf.2018.03.006 30007560 PMC6076350

[14] Gerber Y Weston SA Redfield MM Chamberlain AM Manemann SM Jiang R. contemporary appraisal of the heart failure epidemic in Olmsted County, Minnesota, 2000 to 2010. JAMA Intern Med. (2015) 175:996–1004. 10.1001/jamainternmed.2015.0924 25895156 PMC4451405

[15] Conrad N Judge A Canoy D Tran J Pinho-Gomes A-C Millett ERC. Temporal trends and patterns in mortality after incident heart failure: a longitudinal analysis of 86 000 individuals. JAMA Cardiol. (2019) 4:1102–11. 10.1001/jamacardio.2019.3593 31479100 PMC6724155

[16] Mamas MA Sperrin M Watson MC Coutts A Wilde K Burton C. Do patients have worse outcomes in heart failure than in cancer? A primary care-based cohort study with 10-year follow-up in Scotland. Eur J Heart Fail. (2017) 19:1095–104. 10.1002/ejhf.822 28470962

[17] Park J Bak S Chu H Kang S Youn I Jun H. Current research status and implication for further study of real-world data on East Asian traditional medicine for heart failure: a scoping review. Healthcare. (2024) 12:61. 10.3390/healthcare12010061 38200969 PMC10779411

[18] Ministry of Health and Welfare. Announcement of the 2022 Survey Results on the Utilization of Korean Medicine. Sejong. (2023). Available at: https://www.mohw.go.kr/board.es?mid=a10503010100&bid=0027&tag=&act=view&list_no=375634&cg_code= (accessed 8 August 2024).

[19] Ni Y-M Frishman WH. Acupuncture and cardiovascular disease: focus on heart failure. Cardiol Rev. (2018) 26:93–8. 10.1097/CRD.0000000000000179 29419562

[20] Zhang DY Anderson AS. The sympathetic nervous system and heart failure. Cardiol Clin. (2014) 32:33–45. 10.1016/j.ccl.2013.09.010 24286577 PMC5873965

[21] Lee H Kim T-H Leem J. Acupuncture for heart failure: a systematic review of clinical studies. Int J Cardiol. (2016) 222:321–31. 10.1016/j.ijcard.2016.07.195 27500758

[22] Jung H Yeo S Lim S. Effects of acupuncture on cardiovascular risks in patients with hypertension: a Korean cohort study. Acupunct Med J Br Med Acupunct Soc. (2021) 39:116–25. 10.1177/0964528420920290 32567334

[23] Jia H Lubetkin EI. Life expectancy and active life expectancy by disability status in older US adults. PLoS ONE. (2020) 15:e0238890. 10.1371/journal.pone.0238890 32976543 PMC7518583

[24] Bahk J Kang H-Y Khang Y-H. The life expectancy gap between registered disabled and non-disabled people in Korea from 2004 to 2017. Int J Environ Res Public Health. (2019) 16:2593. 10.3390/ijerph16142593 31330839 PMC6678634

[25] Kim M Jung W Kim SY Park JH Shin DW. The Korea national disability registration system. Epidemiol Health. (2023) 45:e2023053. 10.4178/epih.e2023053 37189275 PMC10482564

[26] (Webpage) Health Insurance Review and Assessment Service standard classification guideline for disease and intervention. Available at: https://opendata.hira.or.kr/op/opc/selectStcPblc.do?sno=13200&odPblcTpCd=004&searchCnd=&searchWrd=&pageIndex=1 (accessed 15 November 2021).

[27] Lee J Lee JS Park S-H Shin SA Kim K. Cohort Profile: The national health insurance service–national sample cohort (NHIS-NSC), South Korea. Int J Epidemiol. (2017) 46:e15. 10.1093/ije/dyv319 26822938

[28] Choi E-K. Cardiovascular research using the Korean national health information database. Korean Circ J. (2020) 50:754–72. 10.4070/kcj.2020.0171 32725984 PMC7441000

[29] Choi JS Kim M-H Kim YC Lim Y-H Bae HJ Kim DK. Recalibration and validation of the Charlson comorbidity index in an Asian population: the national health insurance service-national sample cohort study. Sci Rep. (2020) 10:13715. 10.1038/s41598-020-70624-8 32792552 PMC7426856

[30] Ryu H Jung J Moon J. Patterns of change in cardiovascular risks of Korean male workers: a 10-year cohort analysis using the National Health Insurance Service–National Sample Cohort (NHIS-NSC) 20 database. BMJ Open. (2020) 10:e038446. 10.1136/bmjopen-2020-038446 33154050 PMC7646339

[31] Kim Y-S Kim J Kim Y Kang H-T. Disparities in cause-specific mortality by health insurance type and premium: evidence from Korean NHIS-HEALS cohort study, 2002–2019. BMC Public Health. (2024) 24:1577. 10.1186/s12889-024-19088-3 38867237 PMC11167746

[32] Charlson ME Pompei P Ales KL MacKenzie CR A. new method of classifying prognostic comorbidity in longitudinal studies: development and validation. J Chronic Dis. (1987) 40:373–83. 10.1016/0021-9681(87)90171-8 3558716

[33] Liang B Yan C Zhang L Yang Z Wang L Xian S. The effect of acupuncture and moxibustion on heart function in heart failure patients: a systematic review and meta-analysis. Evid Based Complement Alternat Med. (2019) 2019:6074967. 10.1155/2019/6074967 31772597 PMC6854931

[34] Ganglani V Geng Y-J. The efficacy of acupuncture therapy in the management of dyspnea and other symptoms associated with heart failure: a consolidated review of trial data. Cells Tissues Organs. (2024) 2024:1–24. 10.1159/000539593 38824915

[35] Froehlich-Grobe K Jones D Businelle MS Kendzor DE Balasubramanian BA. Impact of disability and chronic conditions on health. Disabil Health J. (2016) 9:600–8. 10.1016/j.dhjo.2016.04.007 27216441

[36] Kim K. Disability rating system reform in Korea: focusing on improving of the service delivery system for people with disabilities. J Soc Serv Res. (2019) 45:1–11. 10.1080/01488376.2018.1480566

[37] Korea Disabled People's Development Institute. 2015 Yearbook of Disability Statistics. (2015). Available at: www.koddi.or.kr (accessed 4 September 2024).

[38] Wu H Wu J Zhang Z Zheng Y Niu W Zheng L. Prevalence and associated risk factors of hypertension in adults with disabilities: a cross-sectional study in Shanghai, China. Clin Epidemiol. (2021) 13:769–77. 10.2147/CLEP.S322791 34475784 PMC8408044

[39] Jung I Kwon H Park SE Han K-D Park Y-G Rhee E-J. The prevalence and risk of type 2 diabetes in adults with disabilities in Korea. Endocrinol Metab. (2020) 35:552–61. 10.3803/EnM.2020.653 32693567 PMC7520589

[40] Wu J Wang Y Li Y Liu H Yang S Zhai H Wu H. Are physically disabled people at high risk of coronary heart disease among disabled population – evidence from 75-year retrospective cohort study. Ann Epidemiol. (2024) 90:42–8. 10.1016/j.annepidem.2023.11.001 37926391

[41] Martinez-Gomez D Guallar-Castillon P Higueras-Fresnillo S Garcia-Esquinas E Lopez-Garcia E Bandinelli S. Physical activity attenuates total and cardiovascular mortality associated with physical disability: a national cohort of older adults. J Gerontol A Biol Sci Med Sci. (2018) 73:240–7. 10.1093/gerona/glx117 28977342

[42] Wang X Xiao L Xiao L Tian C Liu Y Dai X. The dose-effect relationship of acupuncture on limb dysfunction after acute stroke: a systematic review and meta-analysis. Front Neurol. (2024) 15:1341560. 10.3389/fneur.2024.1341560 38481941 PMC10933065

[43] Yang A Wu HM Tang J-L Xu L Yang M Liu GJ. Acupuncture for stroke rehabilitation. Cochrane Database Syst Rev. (2016) 2016:CD004131. 10.1002/14651858.CD004131.pub3 27562656 PMC6464684

[44] Choi OJ Hwang SY. A comparison of the prevalence of cardiovascular disease and lifestyle habits by disability status and type of disability in Korean adults: a propensity score matching analysis. Res Community Public Health Nurs. (2020) 31:534–48. 10.12799/jkachn.2020.31.S.534

[45] Wang H Lee PMY Zhang J Svendsen K Li F Li J. Association of intellectual disability with overall and type-specific cardiovascular diseases: a population-based cohort study in Denmark. BMC Med. (2023) 21:41. 10.1186/s12916-023-02747-4 36747218 PMC9903576

[46] Zwack CC McDonald R Tursunalieva A Cooray A Lambert GW Lambert EA. Does autonomic nervous system dysfunction influence cardiovascular disease risk in young adults with intellectual disability? Am J Physiol Heart Circ Physiol. (2021) 320:H891–900. 10.1152/ajpheart.00807.2020 33566748

[47] Middlekauff HR Hui K Yu JL Hamilton MA Fonarow GC Moriguchi J. Acupuncture inhibits sympathetic activation during mental stress in advanced heart failure patients. J Card Fail. (2002) 8:399–406. 10.1054/jcaf.2002.129656 12528093

[48] Kimura K Kitagawa Y Tajima F. Effects of a single session of acupuncture treatment on blood pressure and heart rate variability in patients with mild hypertension. J Altern Complement Med N Y N. (2021) 27:342–8. 10.1089/acm.2020.0324 33512256

[49] Bahk J Kang H-Y Khang Y-H. Disability type–specific mortality patterns and life expectancy among disabled people in South Korea using 10-year combined data between 2008 and 2017. Prev Med Rep. (2022) 29:101958. 10.1016/j.pmedr.2022.101958 36161125 PMC9501987

